# A decision tree model for traffic accident prediction among food delivery riders in Thailand

**DOI:** 10.4178/epih.e2024095

**Published:** 2024-11-26

**Authors:** Muslimah Molo, Suttida Changsan, Lila Madares, Ruchirada Changkwanyeun, Supang Wattanasoei, Supa Vittaporn, Patcharin Khamnuan, Surangrat Pongpan, Kasama Pooseesod, Sayambhu Saita

**Affiliations:** 1 Faculty of Public Health, Thammasat University, Lampang, Thailand; 2 Thammasat University Research Unit in Environment, Health and Epidemiology, Pathum Thani, Thailand; 3 Thammasat University Research Unit in One Health and Ecohealth, Pathum Thani, Thailand

**Keywords:** Food delivery riders, Traffic accidents, Decision tree model, Prediction, Mitigation

## Abstract

**OBJECTIVES:**

Food delivery riders (FDRs) play a crucial role in the food delivery industry but face considerable challenges, including a rising number of traffic accidents. This study aimed to examine the incidence of traffic accidents and develop a decision tree model to predict the likelihood of traffic accidents among FDRs.

**METHODS:**

A cross-sectional study was conducted with 257 FDRs in Chiang Mai and Lampang Province, Thailand. Participants were interviewed using questionnaires and provided self-reports of accidents over the previous 6 months. Univariable logistic regression was used to identify factors influencing traffic accidents. Subsequently, a decision tree model was developed to predict traffic accidents using a training and validation dataset split in a 70:30 ratio.

**RESULTS:**

The results indicated that 45.1% of FDRs had been involved in a traffic accident. The decision tree model identified several significant predictors of traffic accidents, including delivering food in the rain, job stress, fatigue, inadequate sleep, and the use of a modified motorcycle, achieving a prediction accuracy of 66.5%.

**CONCLUSIONS:**

Based on this model, we recommend several measures to minimize accidents among FDRs: ensuring adequate sleep, implementing work-rest schedules to mitigate fatigue, managing job-related stress effectively, inspecting motorcycle conditions before use, and exercising increased caution when delivering food during rainy conditions.

## GRAPHICAL ABSTRACT

[Fig f3-epih-46-e2024095]

## Key Message

This study addresses the unmet need for predictive tools to mitigate traffic accidents among food delivery riders (FDRs). Key findings indicate that job stress, fatigue, inadequate sleep, delivering food in rainy conditions, and the use of modified motorcycles significantly elevate the risk of accidents. The decision tree model provided valuable insights for targeted interventions. Scientifically, this model deepens our understanding of the epidemiological factors that influence FDR safety, underscoring the importance of tailored prevention strategies in high-risk work environments.

## INTRODUCTION

The food delivery industry has seen significant growth, primarily fueled by the emergence of online platforms and mobile applications that offer easy access to a wide range of food choices. This shift has reshaped urban mobility and employment landscapes in Thailand. Here, services like GrabFood, foodpanda, and LINE MAN have become essential to urban logistics, ensuring the efficient distribution of food in densely populated areas, particularly during and after the coronavirus disease 2019 (COVID-19) pandemic [1]. Food delivery riders (FDRs) are vital to this ecosystem, acting as the crucial link between consumers and restaurants. However, as they operate within the informal work sector, FDRs in Thailand often do not receive the labor protections and rights that are typically afforded to formal employees [2].

The increasing number of traffic accidents involving FDRs is a troubling trend, with research showing a significant increase in accident rates. For example, a study conducted in Romania observed fluctuations in traffic accidents among these riders, particularly during the COVID-19 pandemic, linking these incidents to unsafe working conditions and high-pressure environments [3,4]. Additionally, a survey in Italy found that 38% of riders had experienced at least 1 accident in the previous year [5]. Previous research has shown that FDRs face unique challenges, including time-sensitive incentives that encourage risky riding behaviors [6]. Psychological factors such as fatigue and stress also play a significant role in mediating the relationship between job demands and traffic [7]. Despite being aware of these risks, riders often place economic survival above safety, navigating dangerous conditions with minimal support from the platforms they work for [8].

Predicting traffic accidents is crucial for improving rider safety and reducing associated risks, as it enables timely interventions and more effective traffic management. Although various factors influencing traffic accidents are recognized, comprehensive models that elucidate the relationship between these factors and accident occurrence are still underdeveloped. Currently, there is a significant gap in the application of machine learning techniques to investigate the factors affecting the likelihood of traffic accidents, especially among informal workers such as FDRs. The J48 algorithm, a decision tree classifier, is a supervised machine learning technique often selected for its interpretability and ease of implementation. It can handle complex relationships and interactions among variables, making it suitable for research that requires explainable models. This is particularly important in fields like public health and safety, where decisions must be comprehensible to stakeholders [9,10]. Therefore, this study aimed to examine the incidence of traffic accidents and to develop a decision tree model specifically designed to predict the likelihood of traffic accidents among FDRs. This model seeks to identify the key factors contributing to accident risks, thereby enabling targeted interventions or strategies to enhance rider safety and reduce the incidence of traffic-related accidents within this vulnerable population.

## MATERIALS AND METHODS

### Study design and study areas

**Figure 1:**
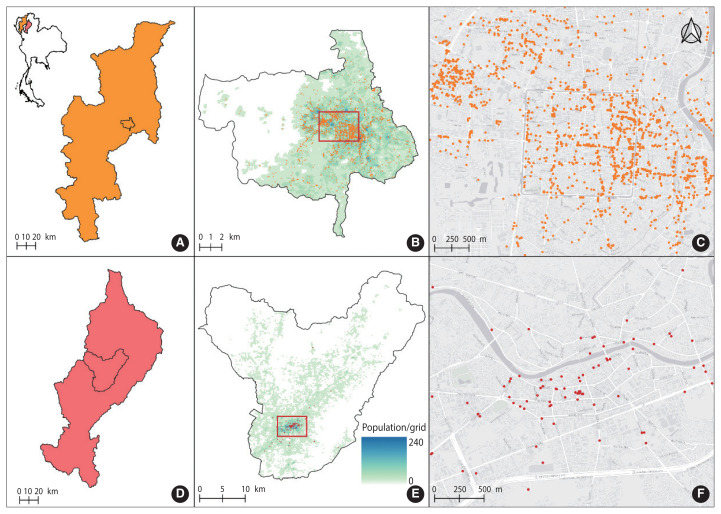
Distribution of food shop locations in Mueang Chiang Mai District (A, B, and C) and Mueang Lampang District (D, E, and F) from OpenStreetMap [11].

This analytical cross-sectional study was conducted in Mueang Chiang Mai District, Chiang Mai Province, and Mueang Lampang District, Lampang Province, located in northern Thailand. According to data from OpenStreetMap (OSM) in August 2023, food-related points of interest (POIs) offer valuable insights into various locations. These include regular and fast food restaurants, bakeries, cafés, and department stores [11], which are frequented by delivery riders who collect food orders to deliver to customers (Figure 1). Most of these establishments support online ordering, a process that typically involves delivery drivers collecting and transporting food to customers.

In Mueang Chiang Mai District (Figure 1A and B), the OSM data reveal a high concentration of food-related POIs in central areas, especially around major roads and downtown locations. Notable clusters are found near landmarks and markets, indicating these areas as prime locations for food shops (Figure 1C). Although food shops are distributed throughout the district, their density diminishes toward the outskirts. In Mueang Lampang District (Figure 1D and E), the OSM data similarly shows a centralized distribution of food-related POIs in the town center and near commercial zones (Figure 1F). The distribution is generally even, though some areas closer to the district boundaries are less densely populated. This centralization minimizes travel time between pickups and deliveries, enabling riders to complete multiple deliveries quickly. Moreover, the strategic placement of food shops along main transportation routes ensures easy access and navigation for delivery riders, thereby enhancing their efficiency and potentially increasing the volume of deliveries. In both districts, the presence of food shops in the outskirts, though sparser, ensures that delivery services can reach a broader customer base, extending food delivery networks into more residential and less commercial areas. This distribution pattern fosters a robust and responsive food delivery system, effectively meeting the needs of customers throughout the districts.

### Study participants

The population of interest in the study comprised FDRs affiliated with major platforms such as GrabFood, foodpanda, and LINE MAN, who are often categorized as informal workers [2]. The sample size for the study was determined based on factors associated with traffic crash experiences identified in a previous study. It was found that riders with over 2 years of experience had 2.59 times the odds of being involved in traffic crashes compared to those with less than 2 years of experience. Additionally, 60.0% of the traffic crash group consisted of riders with more than 2 years of experience (defined as the alternative hypothesis: H_1_), compared to 36.7% among those with less than 2 years of experience (defined as the null hypothesis: H_0_) [12]. To achieve 95% statistical power with a 5% alpha-error probability under a binomial distribution, and using a 2-sided test, the sample size was calculated using G*Power version 3.1.9.7. The initial calculation indicated a need for 238 samples. To account for potential non-responses, the sample size was increased by 10%, resulting in a final sample size of 262. Inclusion criteria required that participants be FDRs with at least 6 months of experience and a willingness to participate.

**Figure 2:**
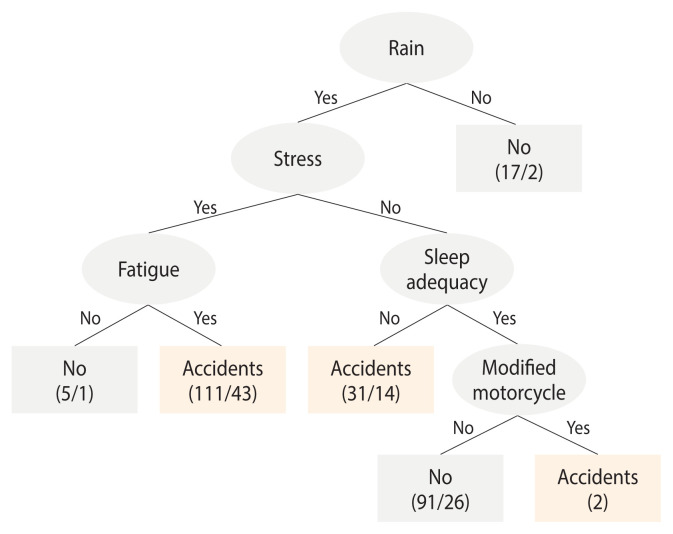
Decision tree model for traffic accidents among food delivery riders.

According to Figure 2, there were differences in the distribution of food shop locations and population density between Muang Chiang Mai and Muang Lampang Districts, which likely influenced the number of FDRs. Consequently, the sample size for Mueang Chiang Mai and Mueang Lampang Districts was set at a ratio of 60:40. Of the 262 participants, 158 were from Mueang Chiang Mai District and 104 from Mueang Lampang District, selected through accidental sampling. However, after verifying the completeness of the data, only 257 questionnaires with complete responses were included in the data analysis for this study.

### Data tools and collection

The data were collected using a structured questionnaire divided into 3 sections: (1) socio-demographic characteristics, which included variables such as sex, age, congenital diseases, education level, source of family income, type of work, and income derived from food delivery riding (with an exchange rate of 1 US dollar approximately equal to 33.50 Thai baht [THB]); (2) work-related factors, which covered work hours, experience in food delivery riding, riding speed, red light running behavior, sleep adequacy, job stress, sources of job stress, fatigue, delivering food during rain, and the use of modified motorcycles; and (3) self-report of accidents and physical pain, which addressed types of accidents, injuries sustained from accidents, and work-related body pains. In this study, “accidents” are defined as any incidents involving FDRs that result in harm or risk while working. These are specifically categorized into slipping (loss of control due to conditions such as wet surfaces), crashing (collisions with vehicles or objects), and scraping (minor abrasions or contact with vehicles or objects). The questions concerning work-related factors and self-report of accidents focused on the challenges faced by FDRs over the past 6 months. Data collection took place between November 2023 and December 2023.

### Statistical analysis

Categorical and numerical variables were described using descriptive statistics, which included numbers, percentages, means, and standard deviations. We compared the socio-demographic and work characteristics associated with having experienced an accident to those of individuals who had never experienced an accident, using an exact-probability test for the categorical variables and t-tests for the numerical variables. To identify factors influencing the occurrence of accidents, univariable logistic regression was conducted. Statistical significance was defined as a p-value of less than 0.05. Data analysis was performed using Stata version 15 (StataCorp., College Station, TX, USA), licensed to the Faculty of Public Health, Thammasat University, Thailand. The decision tree technique was used to integrate all significant variables identified in the univariable analysis.

A decision tree is a method employed in data mining and machine learning to predict the outcomes of various choices by mapping out potential scenarios based on conditions and their probabilities. The J48 algorithm, a specific decision tree technique used in the data mining program WEKA, builds on Ross Quinlan’s earlier ID3 algorithm. This enhancement allows for the creation of decision trees that are not only interpretable but also facilitate a deeper understanding of complex data relationships, thereby supporting informed decision-making based on the resulting models [13]. The J48 algorithm has been applied effectively in analyzing traffic accidents, classifying the severity of these incidents, and predicting outcomes with notable accuracy. Such applications have the potential to enhance traffic safety by reducing the number of accidents and providing insights for initiatives aimed at improving global traffic safety [14,15].

In this study, the J48 algorithm was applied using a 70:30 training-to-validation dataset ratio. The model’s performance was evaluated based on metrics such as accuracy, precision (the percentage of correct predictions out of the total predicted accident cases), and recall (the percentage of correct predictions out of the total actual accident cases). The supervised dataset included instances categorized as “accident” or “no accident,” along with influencing factors identified through univariable analysis. These were used as inputs to construct the model. The aim of this research was to determine a correlation between the presence of influencing factors and the occurrence of traffic accidents. By leveraging this supervised dataset, the model learns to discern patterns and relationships between the influencing factors and the incidence of traffic accidents. This ultimately provides insights into the effectiveness of traffic accident prevention measures among FDRs.

### Ethics statement

The study protocol adhered to the Declaration of Helsinki and received approval from the Institutional Review Board of Boromrajonani College of Nursing, Nakorn Lampang, Thailand, under approval No. E2566-096, dated October 20, 2023.

## RESULTS

### Occurrence of traffic accidents

Among the 257 participants who delivered food using personal motorcycles, 54.9% reported never having experienced a traffic accident, while 45.1% (116 cases) reported involvement in traffic accidents. Of these 116 reported accidents, 43.1% involved slipping, 37.9% involved crashing, and 28.4% involved scraping. Additionally, 83.6% of these cases (97 cases) resulted in injuries (Table 1). All FDRs wore helmets while delivering food.

### Socio-demographic characteristics

A total of 257 FDRs were divided into 2 groups: those who did not report accidents (n=141) and those who did (n=116). The majority of participants were male, with an average age of 34 years. A comparison of the 2 groups showed that the most common characteristics shared by both included being male, aged between 30–45 years, not having a congenital disease, possessing higher education qualifications, being the primary earners in their families, working full-time, and earning a daily income between 500–1,000 THB (p>0.05; Table 2).

### Characteristics of work-related factors

Both groups shared similar work conditions, including adherence to legal speed limits and avoiding running red lights while delivering food. However, they differed in several key areas: experience in food delivery, work hours, sleep adequacy, job stress, fatigue, delivering food during rain, and the use of modified motorcycles. In the accident group, a higher proportion of participants had over 1 year of experience in food delivery, worked more than 8 hours daily (67.2%), experienced inadequate sleep (43.1%), reported job stress (60.3%), felt fatigue (89.7%), delivered food in the rain (98.3%), and used modified motorcycles (12.1%) compared to those in the non-accident group. Additionally, the accident group had higher averages for work hours (10.02±2.91 hr/day) and food delivery experience (28.52±17.31 months) as shown in Table 3. The modifications observed in twenty motorcycles included adjustments to the accelerator or handlebar (40.0%), modifications to the exhaust pipes (40.0%), seat flattening (20.0%), wheel adjustments (20.0%), the addition of thicker seats (10.0%), customized headlights (10.0%), and engine modifications (5.0%).

### Factors influencing traffic accidents

A univariate logistic regression analysis identified several significant variables associated with reported traffic accidents. These include a work-related income of 500–1,000 THB (odds ratio [OR], 1.97; 95% confidence interval [CI], 1.05 to 3.68; p=0.035), food delivery experience of 13–35 months (OR, 2.90; 95% CI, 1.53 to 5.47; p<0.001) and ≥36 months (OR, 2.21; 95% CI, 1.21 to 4.03; p=0.010), working more than 8 hr/day (OR, 1.76; 95% CI, 1.05 to 2.92; p=0.031), inadequate sleep (OR, 1.98; 95% CI, 1.18 to 3.34; p=0.010), job stress (OR, 2.53; 95% CI, 1.53 to 4.19; p<0.001), experiencing fatigue (OR, 2.24; 95% CI, 1.09 to 4.63; p=0.029), delivering food during rain (OR, 6.79; 95% CI, 1.52 to 30.32; p=0.012), and using a modified motorcycle (OR, 3.09; 95% CI, 1.15 to 8.31; p=0.026) (Table 4).

### Prediction model for traffic accidents

The traffic accident prediction model was developed using the decision tree technique, which incorporated various factors that influence traffic accidents. The most effective predictors were identified and applied within the classification tree algorithm. These predictors included delivering food in the rain (labeled as “rain”), job stress, fatigue, sleep adequacy, and the use of a modified motorcycle (Figure 2). The model categorized predictions into 3 scenarios when delivering food during the rain: (1) if the rider was experiencing both job stress and fatigue, the model predicted accidents with 68 correct predictions out of 111 cases (43 incorrect cases); (2) if the rider was not experiencing job stress but was suffering from inadequate sleep, the model predicted accidents with 17 correct predictions out of 31 cases (14 incorrect cases); and (3) if the rider was free from job stress and had adequate sleep, accidents were predicted based on the use of a modified motorcycle, with 2 correct predictions. The model demonstrated a precision of 68.1% and a recall of 66.5%, with an overall accuracy of 66.5%. When analyzed with the validation dataset, the accuracy of the model was 62.3%.

## DISCUSSION

The incidence of traffic accidents among FDRs exhibits substantial variation across different regions and contexts. Previous studies have demonstrated that traffic accidents represent a significant concern, with the number of fatalities among riders increasing during the COVID-19 pandemic, primarily due to increased demand for delivery services and risky riding behaviors [16–18]. This study found that 45.1% of FDRs had been involved in traffic accidents. This trend is consistent with studies conducted in Italy and Vietnam, where 38% and 54% of FDRs, respectively, reported being involved in traffic accidents [5,19]. Although FDRs experienced a moderate rate of slips and crashes, all reported wearing helmets while delivering food. Despite the high proportion of injuries among accident cases (83.6%), other studies have indicated that FDRs exhibit a lower incidence of head and face injuries [20]. These findings underscore the significant traffic safety challenges that a substantial proportion of FDRs face. In this study, the decision tree model developed to predict traffic accidents among food delivery riders identified key risk factors. These include job stress, fatigue, sleep adequacy, delivering during rain, and the use of modified motorcycles.

Job stress significantly influences the occurrence of traffic accidents among FDRs by promoting risky behaviors and impairing cognitive functions essential for safe riding [21,22]. This study identified the top 3 sources of job stress and pressure reported by food delivery workers as insufficient income (63.0%), time pressures (52.5%), and customer expectations (38.1%). FDRs often earn wages that are barely above the poverty line, with some earning 67% below the median income in Italy. This economic vulnerability is compounded by the absence of unionization and inadequate labor protections, which heighten job stress [23]. The instability of income may stem from fluctuating customer demand and the commission-based pay structure, both of which contribute to financial insecurity and increased stress [24]. Time pressure is a significant factor in job stress among food delivery riders. The demand for rapid deliveries frequently results in job overload, intensifying stress levels. This stress, in turn, heightens the likelihood of risky driving behaviors and distractions as riders strive to meet tight deadlines [18,25]. Customer expectations, especially regarding speed and service quality, introduce an additional layer of stress for delivery riders. Direct interactions with customers can sometimes lead to negative experiences, such as encounters with rudeness or even abuse, which significantly affect job stress [26].

In the present study, fatigue emerged as a significant factor in accidents involving FDRs. The prevalence of fatigue among these riders was associated with heightened road safety risks, with a considerable proportion of accidents attributed to fatigue-related issues [5]. Fatigue in FDRs may stem from high job demands, extended work hours, and insufficient sleep. The intense demands of tight delivery schedules and the pressure to meet customer expectations are major contributors to fatigue among FDRs. These demands not only increase fatigue but also impair safety behaviors [27,28]. Furthermore, high job demands are often linked to longer work hours and greater workload intensity, directly increasing fatigue as riders frequently work more than 8 hr/day. A survey in Milan found that 73% of FDRs worked over 8 hours daily and commonly reported fatigue [5]. Moreover, prolonged and continuous physical activity without adequate rest or sleep could lead to muscle fatigue and discomfort [29]. A previous study indicated that most riders sleep less than 8 hours and work more than 8 hours, significantly contributing to their fatigue [30]. The present study found that the FDRs reported muscle fatigue and discomfort, including low back pain (70.8%) and neck and shoulder pain (57.6%). These findings align with the proportions of pain reported among FDRs in the study by Boniardi et al. [5]. The combination of inadequate sleep, excessive work hours, and fatigue plays a crucial role in traffic accidents among FDRs, significantly impairing cognitive functions, reaction times, and decision-making abilities [31–34].

In addition to individual factors, external elements can also influence traffic accidents. Weather is a significant external factor contributing to these incidents. The risk of traffic accidents increases markedly when food is delivered by motorcycle in the rain, primarily due to compromised rider safety [8]. Slippery roads reduce tire traction, making it more difficult to control the motorcycle, especially when braking or turning. Rain further reduces visibility for both riders and other road users. Moreover, the urgency to meet delivery deadlines in poor weather conditions can encourage risky behaviors. This combination of decreased road grip, reduced visibility, and heightened stress significantly raises the probability of accidents and severe injuries [28,35]. Additionally, the demand for food delivery services often rises during adverse weather conditions, such as rain or extreme heat, because people prefer to stay indoors and order online [36]. This increase in demand can perpetuate a cycle of heightened traffic accident risks for FDRs.

The use of modified motorcycles among FDRs was significantly associated with an increased risk of traffic accidents in this study. Modifications included changes to handlebars, exhaust pipes, seats, wheels, headlights, and engines. These modifications could compromise safety standards and alter performance characteristics, such as increased speed and agility, potentially encouraging riders to adopt riskier behaviors [37,38]. Additionally, these alterations might bypass standard safety features, making the motorcycles less stable and reliable, particularly in high-pressure delivery scenarios where speed is often prioritized over caution [39]. Furthermore, previous research indicates that FDRs using modified motorcycles report higher levels of traffic law violations and more frequent collisions compared to those using standard motorcycles [40,41]. Collectively, these factors contribute to a higher incidence of accidents among FDRs using modified motorcycles, thereby exacerbating the hazardous conditions on the road.

The accuracy of the decision tree model, at 66.5%, aligns with results from similar studies, which typically report decision tree accuracies ranging from 65% to 75% [42,43]. This level of accuracy is deemed acceptable for predicting traffic accidents, especially in scenarios where stakeholders require clear and interpretable insights [44]. Additionally, the model achieved a precision of 68.1% and a recall of 66.5%, highlighting its capability to identify critical accident risk factors among food delivery riders effectively. While more complex models, such as random forest, might improve accuracy, they often compromise on interpretability, which is essential for converting findings into actionable safety recommendations [43,45]. Therefore, the decision tree method provides a good balance between predictive performance and clarity, making it suitable for public health applications.

The model in this study effectively categorized traffic accident risks, providing detailed insights into how specific combinations influence the likelihood of accidents. Notably, it indicated that delivering food in rainy conditions, combined with job stress and fatigue, significantly increases the risk of accidents, highlighting the critical impact of psychological and environmental factors on rider safety. Additionally, the model demonstrated that even in the absence of job stress, inadequate sleep can predict accidents with similar accuracy, emphasizing the crucial role of sleep in risk reduction. The inclusion of modified motorcycle use further refined the predictions, particularly when stress and sleep are not dominant factors. This decision tree model underscores the importance of addressing job stress, fatigue, and sleep adequacy in occupational safety protocols for food delivery riders. Recommendations for preventing traffic accidents include ensuring adequate sleep, implementing work-rest schedules to mitigate fatigue, and effectively managing job-related stress. Special caution should also be exercised when delivering food during rainy conditions and when inspecting motorcycle conditions. While the model’s prediction accuracy is moderate, it offers valuable criteria for identifying effective traffic accident prevention strategies among food delivery riders, enabling authorities to implement targeted measures to reduce accidents.

The limitations of this study include the following: (1) The findings may not be fully generalizable to metropolitan areas such as Bangkok, Thailand, characterized by higher levels of urbanization and denser traffic conditions. The distinct traffic dynamics in these regions could result in different accident patterns. (2) The study assesses traffic accident incidence over a 6-month period, which may limit comparability with studies that observe a full year. Factors such as festivals and other seasonal events could influence accident frequency, potentially limiting the study’s ability to generalize findings across an entire year. (3) This study’s reliance on self-reported data introduces potential biases that may affect the accuracy of the results. Recall bias could lead to inaccuracies in participants’ reporting of past events, particularly the conditions surrounding accidents months prior. Additionally, social desirability bias may cause participants to underreport risky behaviors or overreport safety practices, potentially skewing the data towards safer practices [46,47]. Although resource limitations prevented the use of objective measures like Global Positioning System tracking or wearable devices, studies suggest that combining self-reports with objective data sources enhances reliability [47].

## Figures and Tables

**Figure f3-epih-46-e2024095:**
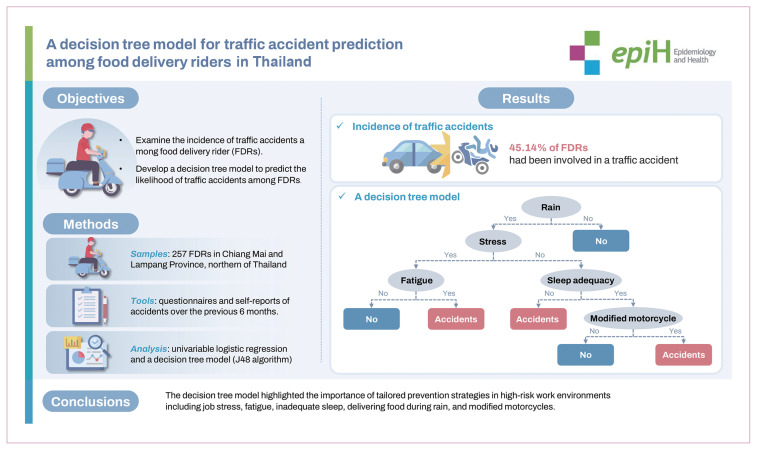


**Table 1:** Occurrence of traffic accidents

Characteristics	n (%)
Traffic accident occurrence	
No	141 (54.9)
Yes	116 (45.1)
Type of traffic accident (n=116)^1^	
Slipping	50 (43.1)
Crashing	44 (37.9)
Scraping	33 (28.4)
Injuries from traffic accident (n=116)	
No	19 (16.4)
Yes	97 (83.6)

1 From multiple options.

**Table 2:** Socio-demographic characteristics of participants who had or had not experienced traffic accidents

Characteristics	Never (n=141)	Accident (n=116)	p-value^1^
Sex			0.214
Female	24 (17.0)	13 (11.2)	
Male	117 (83.0)	103 (88.8)	
Age (yr)			0.505
<30	62 (44.0)	47 (40.5)	
30–45	66 (46.8)	54 (46.6)	
46–60	13 (9.2)	13 (11.2)	
>60	0 (0.0)	2 (1.7)	
Congenital disease			0.314
No	121 (85.8)	94 (81.0)	
Yes	20 (14.2)	22 (19.0)	
Education			0.474
Primary school	5 (3.5)	2 (1.7)	
Secondary school	53 (37.6)	51 (44.0)	
Higher education	83 (58.9)	63 (54.3)	
Being main source of family income			0.375
No	63 (44.7)	45 (38.8)	
Yes	78 (55.3)	71 (61.2)	
Type work			0.081
Full-time	90 (63.8)	86 (74.1)	
Part-time	51 (36.2)	30 (25.9)	
Work-related income (THB)^2^			0.051
<500	38 (27.0)	18 (15.5)	
500–1,000	101 (71.6)	94 (81.0)	
>1,000	2 (1.4)	4 (3.5)	

THB, Thai baht.

1 From exact probability test.

2 1 US dollar ≈ 33.50 THB.

**Table 3:** Characteristics of work-related factors in participants who had or had not experienced traffic accidents

Characteristics	Never (n=141)	Accident (n=116)	p-value^1^
Experience (mo)			0.002
≤12	64 (45.4)	29 (25.0)	
13–35	32 (22.7)	42 (36.2)	
≥36	45 (31.9)	45 (38.8)	
Working hours (hr)			0.040
≤8	65 (46.1)	38 (32.8)	
>8	76 (53.9)	78 (67.2)	
Adhering to legal speed limits (km/hr)			
Yes (≤60)	85 (60.3)	69 (59.5)	0.899
No (>60)	56 (39.7)	47 (40.5)	
Mean±SD	64.89±13.82	65.39±12.36	0.765
Red light running			0.078
No	104 (73.8)	73 (62.9)	
Yes	37 (26.2)	43 (37.1)	
Sleep adequacy			0.012
Yes	102 (72.3)	66 (56.9)	
No	39 (27.7)	50 (43.1)	
Job stress			<0.001
No	88 (62.4)	46 (39.7)	
Yes	53 (37.6)	70 (60.3)	
Fatigue			0.027
No	29 (20.6)	12 (10.3)	
Yes	112 (79.4)	104 (89.7)	
Delivering food during the rain			0.004
No	15 (10.6)	2 (1.7)	
Yes	126 (89.4)	114 (98.3)	
Using a modified motorcycle			0.033
No	135 (95.7)	102 (87.9)	
Yes	6 (4.3)	14 (12.1)	

Values are presented as number (%).

SD, standard deviation.

1 From exact probability test and t-test.

**Table 4:** Factors influencing traffic accidents from univariable logistic regression

Influencing factors	OR (95% CI)	p-value^1^
Work-related income (THB)^2^		
<500	1.00 (reference)	
500–1,000	1.97 (1.05, 3.68)	0.035
>1,000	4.22 (0.71, 25.23)	0.114
Experience with food delivery (mo)		
≤12	1.00 (reference)	
13–35	2.90 (1.53, 5.47)	<0.001
≥36	2.21 (1.21, 4.03)	0.010
Working hours (hr)		
≤8	1.00 (reference)	
>8	1.76 (1.05, 2.92)	0.031
Sleep adequacy		
Yes	1.00 (reference)	
No	1.98 (1.18, 3.34)	0.010
Job stress		
No	1.00 (reference)	
Yes	2.53 (1.53, 4.19)	<0.001
Fatigue		
No	1.00 (reference)	
Yes	2.24 (1.09, 4.63)	0.029
Delivering food during the rain		
No	1.00 (reference)	
Yes	6.79 (1.52, 30.32)	0.012
Using the modified motorcycle		
No	1.00 (reference)	
Yes	3.09 (1.15, 8.31)	0.026

OR, odds ratio; CI, confidence interval; THB, Thai baht.

1 From univariable logistic regression analysis.

2 1 US dollar ≈ 33.50 THB.
